# Sodium Dual‐Ion Batteries with Concentrated Electrolytes

**DOI:** 10.1002/cssc.202201583

**Published:** 2022-09-26

**Authors:** Zhenyu Guo, Gang Cheng, Zhen Xu, Fei Xie, Yong‐Sheng Hu, Cecilia Mattevi, Maria‐Magdalena Titirici, Maria Crespo Ribadeneyra

**Affiliations:** ^1^ Department of Chemical Engineering Imperial College London London SW7 2AZ United Kingdom; ^2^ Department of Materials Imperial College London London SW7 2AZ United Kingdom; ^3^ Key Laboratory for Renewable Energy Beijing Key Laboratory for New Energy Materials and Devices Beijing National Laboratory for Condensed Matter Physics Institute of Physics Chinese Academy of Sciences Beijing 100190 P. R. China

**Keywords:** dual-ion battery, electrochemistry, electrolytes, energy storage, graphite cathode

## Abstract

Na‐based dual‐ion batteries (DIBs) are a class of post‐lithium technology with advantages including extremely fast charging, cost‐effectiveness, and high natural abundance of raw materials. Operating up to high voltages (≈5.0 V), the decomposition of classic carbonate‐based electrolyte formulations and the subsequent fade of capacity continues to be a major drawback in the development of these systems. Here, the performance of a Na‐DIB was investigated in different commonly employed electrolyte system, and a highly concentrated (3 m NaPF_6_) and fluorine‐rich carbonate‐based formulation was optimized to achieve a good performance when compared with literature (based on energy and power density, calculated at coin cell and only using the active mass of active materials).

## Graphical abstract

**Electrolytes**: Na‐based dual‐ion batteries (Na‐DIBs) operate up to high voltages (≈5.0 V), but the decomposition of carbonate‐based electrolyte formulations is a major drawback in the development of these systems. Herein, the performance of a Na‐DIB in different electrolyte systems is investigated, and a highly concentrated (3 m NaPF_6_) and fluorine‐rich carbonate‐based formulation is optimized for good performance.

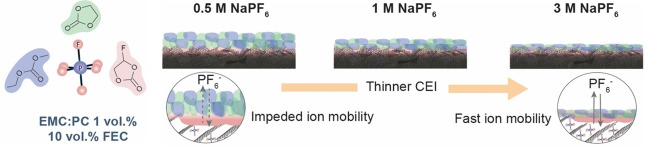

## Introduction

The global objective of achieving carbon neutrality by 2050 calls for greater diversification of energy storage chemistries, beyond Li‐ion batteries. Current Li‐based technologies rely on critical or expensive raw materials, with largely negative socio‐environmental impacts of extraction and high risk of supply disruption.^[1]^ To design a better future, post‐lithium technologies must target higher capacities, faster charging, longer lifetimes, and decreased operational hazards, both in an economical and sustainable manner.[^1b^, ^2^] In this context, Na‐based dual‐ion batteries (DIBs) are a promising alternative since Na is low‐cost ($150 ton^−1^ vs. $5000 ton^−1^ for Li), has a low electrochemical potential (−2.71 V vs. standard hydrogen electrode), and is abundant (2300 ppm vs. 18 ppm for Li) and evenly distributed across the globe.^[3]^ Additionally, the cathode in DIBs is normally graphite, which eliminates the need for critical metals (e. g., Ni, Co) and reduces the manufacturing cost.

**Figure 1:**
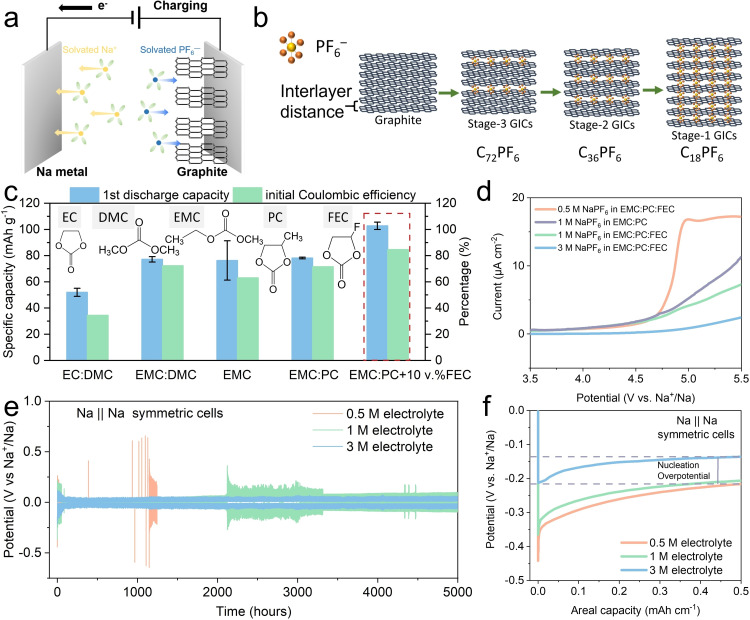
(a) Schematic diagram of the working principle of a DIB of a configuration of Na metal||graphite cathode upon charging. (b) Schematic diagram of a staging intercalation model from graphite to stage‐1 graphite intercalation compound. (c) Column chart summarizes the averaged 1st initial discharge capacity and the highest initial coulombic efficiency from three parallel coin cells using 1 m NaPF_6_ in different solvents (insets show the chemical structures of the different solvents employed). (d) Linear sweep voltammetry (LSV) up to 5.5 V vs. Na^+^/Na at a sweep rate of 1 mV s^−1^ to assess the onset potential at which the different electrolytes start degrading. (e) Long‐time cycling stability of electrolytes of different concentrations in symmetric Na||Na cells, striping and plating at a current density of 1 mA cm^−2^ with a specific capacity up to 0.5 mAh cm^−2^. (f) 1st cycle potential–capacity profiles of Na||Na symmetric cells using 0.5, 1, and 3 m NaPF_6_ in EMC/PC (1 : 1 *v*/*v*) with 10 vol% FEC at a current density of 1 mA cm^−2^ with a specific capacity up to 0.5 mAh cm^−2^.

In DIBs, both the anions and cations in the electrolyte (e. g., Na^+^ and PF_6_^−^) play an active role: upon charging, (Figure 1a) anions intercalate at the cathode and cations are reduced at the anode (e. g., Na^+^→Na^0^).^[4]^ The intercalation of PF_6_^−^ within graphite has been proposed to evolve through several intercalation compounds (GICs): stage‐3 or the formation of C_72_PF_6_, stage‐2 or formation of the C_36_PF_6_, and stage‐1 or formation of the C_18_PF_6_ GIC (Figure 1b).^[5]^ This staggered intercalation working principle enables shorter and less tortuous diffusion pathways for ions; thus, DIBs are characterized by fast charging and high cut‐off voltages of 5.0 V.

Based on the above merits, DIBs are ideal for scenarios where cost‐effectiveness and high‐power energy storage are required, even if the theoretical capacity of graphite is limited (≈100 mAh g^−1^). However, the fundamental challenge is that standard electrolytes for Li‐ion batteries [e. g., 1 m LiPF_6_ in ethylene carbonate (EC) and diethyl carbonate] are inherently incompatible with DIBs. For example, EC, which normally enables a uniform and protective solid electrolyte interphase (SEI) layer in LIBs,^[6]^ solvates PF_6_^−^ anions too strongly,^[7]^ inhibiting its intercalation into graphite and reducing the plateau capacity.^[8]^

Alternative electrolyte formulations are still under development,^[9]^ and systematic studies toward a better understanding of the SEI in these systems are scarce.[^4^, ^10^] To address this gap, in this work various electrolyte combinations, varying the salt concentration, additives, and the nature of the solvent, were screened. The best system was found to be 3 m NaPF_6_ in an ethyl methyl carbonate/propylene carbonate (EMC/PC, 1 : 1 *v*/*v*) solution and with 10 vol% fluoroethylene carbonate (FEC) as additive. By employing this electrolyte, a Na||graphite cell can deliver a discharge specific capacity of 92.7 mAh g^−1^ at 2.0 A g^−1^ (the current and the capacity were based on the mass of carbon active material at coin cell level). Through depth profiling X‐ray photoelectron spectroscopy (XPS) we were able to determine that with higher concentration of electrolyte a thinner and more protective SEI is formed on the graphite cathode (cathode electrolyte interphase, CEI). This thin layer enables fast diffusion kinetics, and therefore the migration of ions is not compromised at high current densities. Since at these high energy densities the inhomogeneous plating/stripping of Na inevitably induces the formation of dendrites and affects the battery lifetime, we also present a lignin‐derived carbon nanofiber mat as a metallic sodium host. Our all‐carbon dual‐ion full cell (Na‐plated carbon fiber mat||graphite) uses only the minimum amount of sodium and exhibits a specific discharge capacity of 66.3 mAh g^−1^ at a current density of 1.6 A g^−1^ (the current and the capacity were based on the mass of both the anode and cathode at coin cell level).

## Results and Discussion

Highly crystalline graphite (see Figures S1–S4 and Table S1 for more details on morphology and texture) with an average particle size of 9–10 μm in length was selected as the cathode material for its low aspect ratio (length/width/height≈1 : 1.12:0.3) and high graphitization degree, as this has been proved to enable reaching higher specific discharge capacities.^[11]^ Considering that ions start intercalating from the graphite edges,^[12]^ small but thick particles (e. g., high edge‐to‐basal‐plane ratio) guarantee a larger amount of shorter graphite interlayers available for the ions to intercalate.^[13]^

For the electrolyte system, 1 m NaPF_6_ was selected as the salt since the fluorine from the anion can contribute to stabilizing the SEI formation on electrodes even at high voltages.^[14]^ Additionally, graphite can accommodate PF_6_^−^ efficiently, and NaPF_6_ is also the most economically viable electrolyte for commercial Na‐ion batteries nowadays.^[15]^

Regarding the solvent, as shown in Figure 1c (see additional results in Figure S5) linear and cyclic carbonates were screened [dimethyl carbonate (DMC), EMC, PC FEC] as well as their mixtures (EC/DMC, EMC/DMC, EMC/PC) in addition to two different concentrations of FEC as a F‐providing additive (EM/PC+20 vol% FEC and EMC/PC+10 vol% FEC). These electrolyte systems were tested in Na||graphite cells and compared in terms of their initial discharge capacity and initial coulombic efficiency.

At this concentration (1 m), the performance is determined by the complex interplay between conductivity, viscosity, contact ion pair interactions, solvation (e. g., donor and acceptor numbers), and redox potential of the electrolyte. The EC/DMC starting choice displayed the lowest discharge capacity, while replacing EC with EMC enabled increasing both the initial coulombic efficiency and the initial discharge capacity. A plausible explanation for this behavior is that EC, a cyclic solvent with extremely high dielectric constant (*ϵ*_EC_=90.5 at 40 °C), would tend to form small clusters (≈1 nm) in the mixture with the low‐*ϵ* DMC (*ϵ*_DMC_=3.1 at 25 °C),^[16]^ while the mixture of DMC and EMC (*ϵ*_EMC_=2.9 at 25 °C) will show a better dielectric affinity. In the low dipolar EMC/DMC mixture, even if the formation of contact ion pairs should be in principle more probable, for NaPF_6_ this effect will be thermodynamically less pronounced than for other salts due to the low donor number of PF_6_^−^ ions (DN_PF6−_=2.50).

The effect of the solvent characteristics is also reflected in the ability of PF_6_^−^ to intercalate; the more freely the ions move, the less energy will be needed to fill the graphite cathode. In the case of the non‐ideal EC/DMC mixture, full PF_6_^−^ intercalation is not reached, as the lowest voltage plateau capacity, attributed to the stage‐1 GICs,[^3a^, ^17^] is not observed (see Figure S6 in the Supporting Information). For the EMC/DMC mixture, the reduction in viscosity and the lower solvation energy of both solvents might be responsible for better PF_6_^−^ intercalation, and thus an improved capacity and reversibility.^[18]^ EMC has indeed been suggested as one of the best co‐solvents to facilitate PF_6_^−^ intercalation into graphite due to improved ion mobility (higher ionic conductivity) and a certain capacity to act as a “lubricant” for the insertion/extraction of PF_6_^−^.^[19]^ However, EMC permittivity is too low to perform satisfactorily on its own, and as a consequence, the initial coulombic efficiency and the initial discharge capacity did not show an improvement in comparison to EC/DMC. The addition of PC to EMC, with a high dielectric constant yet a lower DN than EC (DN_PC_=15.1 vs. DN_EC_=16.4) ensures better solvent co‐solubility, lower ion‐pair interactions, and higher ionic conductivity, giving rise to a balanced solvation/de‐solvation energy to enable optimal PF_6_^−^ intercalation into graphite.[^9b^, ^10^, ^18^] As such, the EMC/PC mixture shows improved initial coulombic efficiency and initial discharge capacity than both EMC or PC on their own. However, when testing the electrochemical stability of 1 m NaPF_6_ in EMC/PC (Figure 1d and Figure S7), despite being more stable than the classic formula (1 m NaPF_6_ in EC/DMC), the onset voltage for decomposition is only around 4.6 V. Increasing the stability of the electrolyte is important to increase the safety of DIBs, as the high cut‐off voltage electrolyte decomposition can induce gas formation, corrosion, and battery explosion.^[20]^ The addition of 10 vol% FEC, which decomposes prior to the solvent, enables passivating the polarizable electrode interphase,^[21]^ and consequently, the onset voltage of the 1 m EMC/PC with 10 vol% FEC electrolyte increased to around 4.7 V. However, further broadening of the window was only possible when the concentration of NaPF_6_ was increased to 3 m. Decreasing the number of free solvent molecules decreases their chance of decomposing electrochemically^[22]^ and also increases the energy density by using less amount of electrolyte.^[23]^ The same positive effect of increasing the concentration of NaPF_6_ was observed when the electrolytes were tested in symmetric Na||Na coin cells (Figure 1e and Figure S10). The 3 m cell can cycle stably for more than 5000 h of charge/discharge, while the 0.5 and 1 m cells failed earlier, proportionally to the concentration of salt in the electrolyte. When the nucleation overpotential during the first cycle was evaluated in these cells (Figure 1f), the 3 m electrolyte showed the lowest value (76.6 mV vs. 159 mV for 1 m and 225.7 mV for 0.5 m). This suggests that at higher electrolyte concentration, the ionic sodium would be more homogeneously plated/stripped from the metallic Na surface, explaining the better durability of the symmetric cell at 3 m.

**Figure 2:**
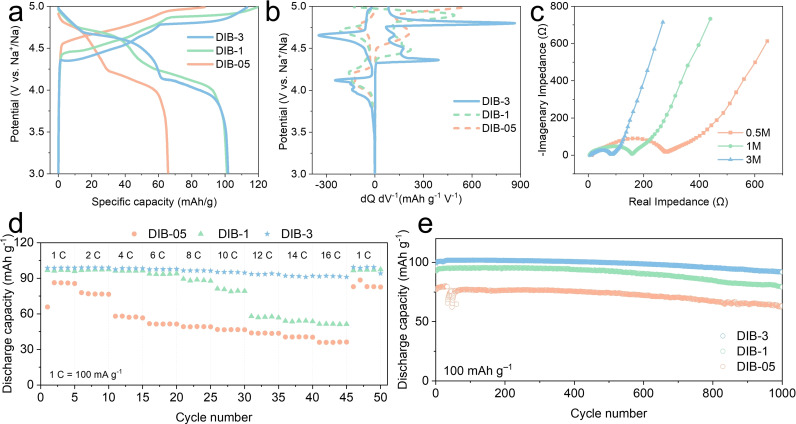
(a) 1st cycle of the potential–capacity profiles of DIB‐05, DIB‐1, and DIB‐3 at a current density of 100 mA g^−1^ in a voltage window of 3.0–5.0 V (V vs. Na^+^/Na). (b) Differential capacity d*Q* d*V* ^−1^ plot derived from the 1st cycle potential–capacity profiles. (c) Electrochemical impedance spectroscopy (EIS) (Nyquist plot) of graphite cathodes cycled after 10 cycles at 100 mA g^−1^ at 5 V. (d) Rate performance from 1 to 16 C (1 C=100 mA g^−1^) of DIB‐05, DIB‐1, and DIB‐3. (e) 1000‐cycling performance at 100 mA g^−1^ of DIB‐05, DIB‐1, and DIB‐3.

To further explore the influences of the electrolyte concentration on the electrochemical performance of DIBs, the three electrolyte concentrations [3, 1, and 0.5 m NaPF_6_ in EMC/PC (1 : 1 *v*/*v*)+10 vol% FEC] were used to assemble Na||graphite DIBs; these were denoted as DIB‐3, DIB‐1, and DIB‐05, respectively. Figure 2a shows the 1st galvanostatic charge/discharge profile for the three systems, and Figure 2b shows their differential capacity (d*Q* d*V* ^−1^) plot. The d*Q* d*V* ^−1^ plot is normally used to evidence the plateau regions. A sharp peak in differential capacity corresponds to a plateau region in the galvanostatic profile. By increasing the concentration from 1 to 3 m, the initial coulombic efficiency increases from 84.8 to 90.0 % (Figure S9). DIB‐3 shows a distinct charge plateau located between 4.77–4.80 V, lower than that of DIB‐1 (4.85–4.90 V) and DIB‐05 (4.95 V). Nearly half of the capacity is available in the high‐voltage plateau region, adjoining the cut‐off voltage.

The Nyquist plots after 10 cycles (Figure 2c) show an obvious trend: the higher the electrolyte concentration the lower the charge‐transfer resistance, as evidenced by the smaller radius of the first semicircle when concentration increases (DIB‐3<DIB‐1<DIB‐05). Figure 2d shows the rate performance for each concentration at varying current densities from 1 to 16 C (1 C=100 mA g^−1^). DIB‐3 and DIB‐1 deliver comparable discharge capacities at low current densities up to 600 mAh g^−1^, while DIB‐3 outperforms DIB‐1 and DIB‐05 above 800 mA g^−1^. This can be mainly attributed to the low overpotential and low charge‐plateau of DIB‐3. The overpotential on the Na metal anode, particularly at high rates, can cause the cells to reach the cut‐off potential prematurely,^[24]^ preventing them from fully charging/discharging. This effect is larger for DIB‐05 and DIB‐1, as they present higher voltage plateaus and higher cell resistance, as shown in the impedance plots. Hence, the lower charging plateau of DIB‐3 is beneficial to enable reaching high power density. After 1000 cycles (Figure 2e) the capacity retention was 92, 83, and 85 % for DIB‐3, DIB‐1, and DIB‐0.5, respectively, corroborating the excellent cyclability and performance achieved through increasing the concentration of NaPF_6_ in the optimum EMC/PC electrolyte with FEC additive.

**Figure 3:**
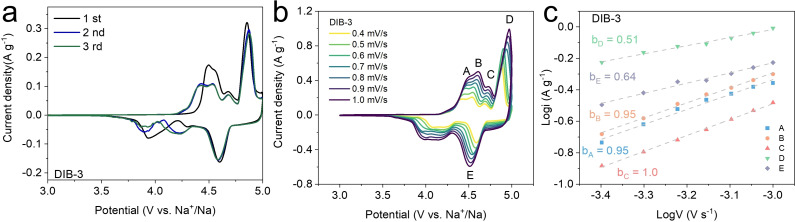
(a) First 3 cycles of CV curves of the DIB‐3 at a scan rate of 0.2 mV s^−1^. (b) CV scans of the DIB‐3 at varying scan rates from 0.4 to 1.0 mV s^−1^. (c) Calculated *b* values of the DIB‐3 from the selected five peaks and their fitted lines.

To study the intercalation of PF_6_^−^ in more detail, the first three cyclic voltammetry (CV) scans were evaluated. By combining the differential capacity from Figure 2b and CV curves (see Figure 3a for the first three CV curves of DIB‐3), the working voltage of the DIB‐3 can be extracted (between 4.32 and 5.0 V). The 1st CV curve of DIB‐3 does not overlap with the rest of the cycles since the onset potential in the 1st cycle is around 4.35 V, which is higher than 4.2 V, the onset for the rest of the cycles. During the 1st cycle, graphite has its native interlayer distance; therefore, the large PF_6_^−^ anions need to overcome a very high energy barrier to start intercalating. From the 2nd cycle, since partial exfoliation and entrapped PF_6_^−^ ions have already widened the interlayer distance, graphite does not behave as pristine but rather as expanded graphite. Thus, subsequent intercalation is more favorable and onsets at lower potentials (see Figure 3a and Figure S8 for the first three CV curves of DIB‐3, DIB‐1, and DIB‐05). To understand the ion storage mechanism of DIB‐3, CV scans at a varying scan rate of 0.4 to 1.0 mV s^−1^ were performed. From Figure 3b, five CV peaks (A–E) were selected to study the kinetics behind the PF_6_^−^ intercalation stages within graphite (from stage 5 to 1, each number denoting the number of graphene layers intercalated between GICs).

Faster CV scan rates lead to a thinner diffusion layer, and therefore, higher currents are observed.^[25]^ Using the relationship between the scan rate (*v*) and the peak current (*i*) (*i*=*av* ^*b*^), Figure 3c shows the slope (*b*) values for each electrochemical process. The oxidative scans show four peaks (A–D), representing different intercalation stages. This section, between 4.25 and 4.75 V, corresponds to the sloping region, while above 4.78 V (D peak), the process is linked to the plateau region. This latter exhibits the highest current density of all the scan rates since it is related to the main intercalation process. The calculated *b* values for peaks A, B, and C are all close to 1 so that the currents are nearly linear to the scan rate. This suggests the sloping region is mainly dominated by a pseudo‐capacitive regime, in which surface reactions take place predominantly. The *b*_D_ value of 0.51, on the other hand, suggests that intercalation (plateau region) is mainly controlled by the semi‐infinite diffusion of ions. Therefore, stage‐1 GICs are being formed and the battery is able to reach the full depth of charge/discharge.

In the case of the reduction scans, E peaks give rise to a *b*_E_ value of 0.64, corresponding to the discharge plateau at around 4.6 V. This process is more likely to be diffusion‐controlled, yet with a large pseudo‐capacitive contribution. For intercalation to continue proceeding, subsequent PF_6_^−^ anions need to overcome larger energy barrier of both the repulsion forces from intercalated PF_6_^−^ anions and the van der Waals forces from the π–π graphite stacks. In contrast, for de‐intercalation, both forces can instead energetically facilitate the extraction of PF_6_^−^ anions. This is evidenced through the larger value of *b*_E_ (faster de‐intercalation process) in comparison to *b*_D_.

**Figure 4:**
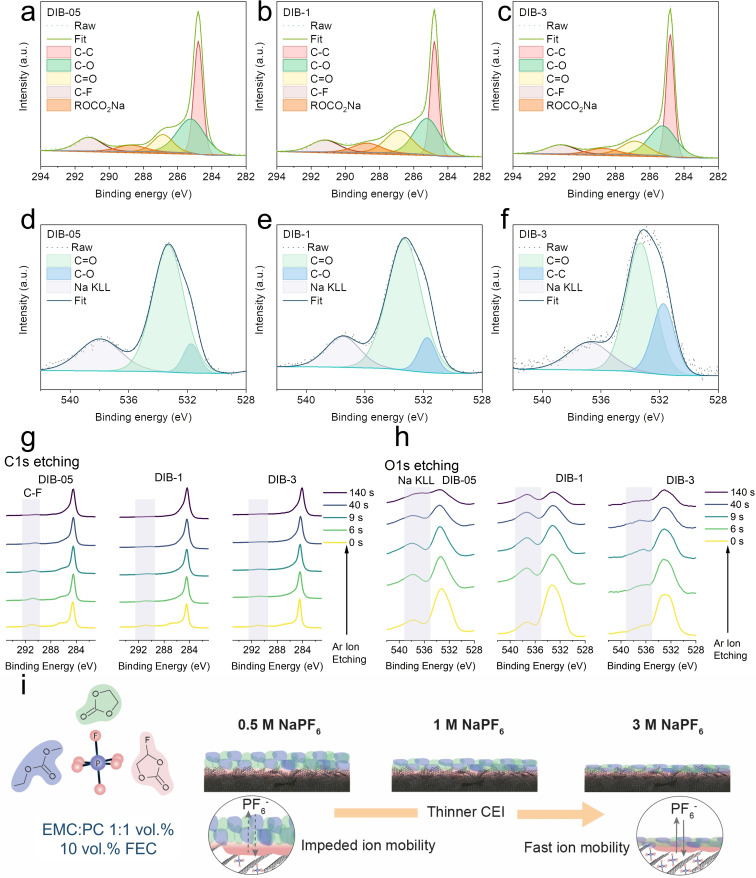
Fitted C 1s core level XPS spectra of (a) DIB‐05, (b) DIB‐1, and (c) DIB‐3 after the first 5 cycles at 100 mA g^−1^. Fitted O 1s core level XPS spectra of (d) DIB‐05, (e) DIB‐1, and (f) DIB‐3 after the first 5 cycles at 100 mA g^−1^. (g) Depth profile of the C 1s XPS spectra of DIB‐05, DIB‐1, and DIB‐3. (h) Depth profile of the O 1s XPS spectra of DIB‐05, DIB‐1, and DIB‐3. (i) Schematic diagram of the CEI layer induced by electrolytes with different concentrations.

Ex‐situ X‐ray photoelectron spectroscopy (XPS) was carried out to study the composition of the cycled graphite cathodes. The high‐resolution C 1s (282–294 eV) core‐level spectra (Figure 4a–c) of all systems (DIB‐05, DIB‐1, and DIB‐3) were fitted using five chemical environments: C−C (284.8 eV), C−O (285.2 eV), C=O (286.9 eV), C−F (291.2 eV), and ROCO_2_Na (288.7 eV).[^3g^, ^4^, ^10^] For DIB‐05 and DIB‐1, the C−O and C=O contribution was larger (45.98 % for DIB‐05 and 52.26 % for DIB‐1 vs. 45.66 % for DIB‐3, see Tables S2 and S3 for more detail), due to the higher probability of solvent‐ion interactions when the concertation of the salt is lower. As the PF_6_^−^ anion reaches the graphite cathode, a certain degree of de‐solvation prior to intercalation occurs. The de‐solvated molecules then decompose oxidatively into polyesters (traced through the C=O and C−O XPS peaks^[10]^), however, at the highest salt concentration (3 m, DIB‐3) this seems to be suppressed. The O 1s band (Figure 4d–f), fitted using C=O (533.2 eV), C−O (531.6 eV), and Na KLL (537.9 eV), follows the same trend that C 1s (higher C=O contribution for DIB‐05 and DIB‐1)^[3f]^ corroborating the greater oxidative decomposition of the carbonate solvent at lower salt concentrations.

To have a clearer understanding of the differences across the CEI, the cycled electrodes were progressively Ar^+^‐etched and a compositional depth profile was obtained by monitoring the C 1s and O 1s spectra at different etching times (6, 9, 40, and 140 s, Figure 4g,h).^[7]^ The sp^2^ carbon environment of the C 1s band is predominantly attributed to the pristine hard carbon, which is partially buried underneath the CEI after cycling. With etching time, the percentage of the sp^2^ contribution increases for all electrodes whilst the C−F line (291.2 eV) disappears. The prominence of this line is greater when the electrode is cycled in the lowest NaPF_6_ concentration (DIB‐05) while at 3 m (DIB‐3) it is almost imperceptible even before etching (*t*=0 s). This suggests that the CEI is thinner for the electrolyte with a smaller number of free solvent molecules available for electrochemical degradation. A thinner CEI layer has lower resistance and higher ionic conductivity, in accordance with the electrochemical impedance spectroscopy (EIS) results in Figure 3c, which facilitates ion diffusion, especially at a high current rate.

For the sake of comparison, a cycled graphite electrode, charged/discharged for 5 cycles in the 0.5 m NaPF6 electrolyte, was disassembled, washed with a mixture of EMC/PC/FEC, and subsequently assembled into a DIB using the 3 m electrolyte. No improvement in cycling stability or rate performance was observed, corroborating that a thick CEI layer is irreversibly formed during the first charge/discharge cycles when using a less concentrated electrolyte; once the CEI is formed, ion transport is impeded to a greater extent if the interphase is thicker, leading to poor rate performance and eventual capacity fading.

**Figure 5:**
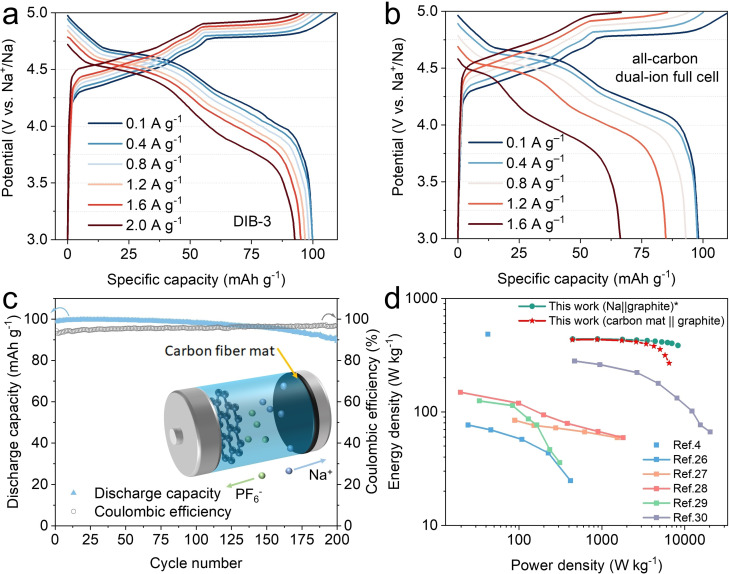
(a) Potential‐capacity profiles of the DIB‐3 at increasing current densities from 100 mA g^−1^ to 2.0 A g^−1^. (b) Potential‐capacity profiles of the all‐carbon dual‐ion full cell at increasing current densities from 100 mA g^−1^ to 1.6 A g^−1^. (c) 200 cycles performance at 100 mA g^−1^ showing 90 % capacity retention of an all‐carbon dual‐ion full cell with a configuration of carbon fiber mat anode|3 m NaPF_6_ EMC/PC/FEC|graphite cathode; the inset is a schematic diagram of the all‐carbon dual‐ion full cell upon charging. (d) Ragone plot comparing different sodium‐based battery technologies.[^4^, ^26^, ^27^, ^28^, ^29^, ^30^] * indicates that in the plot for Na||graphite DIBs, the energy/power density were calculated based on the mass of graphite cathode only at a coin cell level.

The galvanostatic charge/discharge profiles of the Na||graphite DIB‐3 at different current densities [between 100 mA g^−1^ (1 C) and 2.0 A g^−1^ (20 C)] are shown in Figure 5a. DIB‐3 in the optimized electrolyte system can deliver more than 92 % capacity (92.7 mAh g^−1^) at 2 A g^−1^ and around 100 mAh g^−1^ at 100 mA g^−1^. The main high‐voltage charge plateau is slightly shortened, as the onset voltage increases from around 4.78 to 4.90 V with increasing charge current, which is caused by the polarization of the Na metal. At a current density of 100 mA g^−1^ (1 C, charge time ≈1 h) the power density is 440 W kg^−1^ for discharge for an energy density of 439 Wh kg^−1^. At the much higher charging rate of 2.0 A g^−1^ (20 C, charge time ≈2.78 min), the maximum discharge power density is 8296 W kg^−1^ for an energy density of 385 Wh kg^−1^. These values are only based on the weight of graphite and at the coin cell level. Based on these results, DIB‐3 maintains a high energy density (87.7 %) between the entire current density window (100 mA g^−1^ to 2.0 A g^−1^) while it achieves an 18‐fold increase in power density. When only based on the mass of active materials for comparison with similar calculations in the literature, our results are promising for energy and power density of batteries and outperform a multitude of results reported in the literature, as shown in the Ragone plot in Figure 5d.

Considering the practicalities of manufacturing DIBs, it would be unrealistic and unsafe to have a commercial battery with bare Na metal as the anode. We therefore propose an all‐carbon dual‐ion full cell by incorporating a sustainable (lignin‐derived) carbon fiber mat as a conductive and 3D host for Na. We employed this Na/C composite as the anode vs. graphite in the optimized electrolyte used for DIB‐3. The fiber mat is ultralight (<0.05 mg cm^−2^) and contains a high proportion of oxygen functionalities that can improve its sodiophilic character^[3g]^ to enable homogenous Na plating/stripping. The carbon fiber mat was galvanostatically pre‐plated with Na (see the Experimental Section and Figure S11 for details) and transferred into a fresh DIB with a graphite cathode. The boundaries of the charge/discharge plateaus (Figure 5b) remained unaltered, indicating that the polarization and nucleation of the Na on the mat do not negatively influence or retard the insertion/extraction of ions from the electrodes. Our all‐carbon DIB concept not only uses a sustainable fiber mat to protect the metallic Na but also retains an extremely high power density. For the carbon fiber mat, the galvanostatically pre‐plated Na amount is rather low, and therefore the active area and available sodium are lower than in the half‐cell configuration using Na‐metal. This could trigger a lower rate capacity (Figure 5b) in comparison to the half‐cell configuration (Figure 5a). Although in terms of cycling stability (Figure 5c) and rate performance (Figure 5b) the all‐carbon DIB is outperformed by the DIB employing a Na metal anode, at a high current density (1.6 A g^−1^) it still delivered a discharge power density of around 6494 W kg^−1^ at 269 Wh kg^−1^ (the current and the capacity were based on the mass of both electrodes at coin cell level only). We believe our concept paves the way for accelerating the market uptake of next‐generation Na‐based DIBs. Although the gravimetric power and energy density are remarkable, both the volumetric energy and power density are rather modest. Here, the volumetric data is reported merely for comparing it with literature values; however, work to address the limitation of the large volume of the graphite cathode is ongoing. Further optimization of the electrolyte system and monitoring of each intercalation/extraction stage in relation to capacity retention over time is currently in progress to enable further understanding of these fascinating systems.

## Conclusions

This work achieves a stable and sustainable Na‐based dual‐ion battery (DIB) with a high‐power density. We screened a range of solvents and concentrations for DIBs and rationally optimized an efficient electrolyte formulation: 3 m NaPF_6_ ethyl methyl carbonate/propylene carbonate (EMC/PC, 1 : 1 *v*/*v*) with 10 vol% fluoroethylene carbonate (FEC). Due to improved oxidative stability and enhanced intercalation capacity of concentrated electrolyte, a proof‐of‐concept DIB using a 3 m electrolyte delivered a higher power density and longer cycling performance. Ex‐situ etching X‐ray photoelectron spectroscopy showed that 3 m electrolyte forms the thinnest cathode electrolyte interphase layer on a graphite cathode, which facilitate fast charging. A concept of the all‐carbon dual‐ion full cell was also demonstrated.

## Experimental Section

### Materials synthesis

The graphite powder (46304 Graphite powder, synthetic, APS 7‐11 micron, 99 %) was purchased from Alfa Aesar. Sodium metal, 1‐methyl‐2‐pyrrolidinone solution (NMP, anhydrous, 99.5 %), and poly(vinylidene fluoride) binder (PVDF, average *M*_w_≈534000 by gel permeation chromatography, powder) were purchased from Merck. NaPF_6_ (99.0 %), EMC (99.99 %), PC (99.99 %), and FEC (99.9 %) solvents were purchased from Guangdong Canrd New Energy Technology Ltd. CR2032 coin cells and parts were purchased from both Pikem and Guangdong Canrd New Energy Technology Ltd.

### Electrochemical measurements

For graphite cathode coating slurry, 90 wt % graphite powder and 10 wt % PVDF were mixed in an NMP solution. The slurry was then coated onto Al foils (16 μm in thickness), followed by drying in a ventilated oven at 80 °C overnight before cutting into small electrodes (1 cm in diameter). An argon‐filled glovebox (H_2_O<0.5, O_2_<0.5) was used when assembling the coin cells. DIBs using different electrolytes were assembled in the same manner (e. g., mass loading of 2 mg cm^−2^, same pressure of 70 psi, glass fiber A as the separator, Na metal of 1.2 cm in diameter). A piece of metallic Na (12 mm in diameter) was used as both the counter electrode and anode. Glassy fiber (Whatman, class A) was used as the separator soaked with 80 μL. The 0.5, 1.0, and 3.0 m NaPF_6_ in EMC/PC (1 : 1 *v*/*v*) with 10 vol% FEC were selected as the electrolytes. All the DIBs were discharge/charged between 3.0–5.0 V (vs. Na^+^/Na). Galvanostatic charge and discharge, rate performance, and long cycling were performed on a LAND CT2001A battery testing system. CV and LSV were performed on a Biologic potentiostat system. EIS from 100 kHz to 0.01 Hz was performed on a Biologic potentiostat system.

To test the stability of electrolytes, coin cells with Na metal against a stainless‐steel spacer were assembled and tested using LSV at a scan rate of 1 mV s^−1^ up to 5.5 V (vs. Na^+^/Na).

Pre‐plating took place in a half cell at a discharge current density of 1 mA cm^−2^ for 1 h in the 3 m electrolyte; the amount of pre‐plated Na was controllable by adjusting the discharge time.

### Structural characterization and analysis

The average size of graphite was calculated using selected 20 graphite particles from the scanning electron microscopy (SEM) image in Figure S3. The size (length/height/thickness) was measured and averaged by using the ImageJ software. Raman spectroscopy was performed on a Renishaw Raman instrument using a 532 nm laser. The morphology and shape of samples were obtained using SEM, Zeiss Auriga Cross Beam. X‐ray diffraction (XRD) measurement was carried out by an X'Pert PRO PANalytical (40 mA and 40 kV for power settings). The Brunauer–Emmett–Teller (BET) method and density functional theory (DFT) were used to evaluate the surface area and pore size distribution from the N_2_ adsorption/desorption isotherm by a physisorption instrument (Micromeritics 3 Flex).

### Ex‐situ XPS electrode preparation

XPS was conducted using a Thermo Fisher K‐Alpha^+^ XPS facility, and data were fitted using the Avantage software. The graphite electrodes for XPS measurement were prepared by cycling at 100 mA g^−1^ for 5 cycles between 3.0–5.0 V before dissembling, then washed using EMC/PC/FEC mixed solvent in a glovebox to minimize the amount of NaPF_6_ salt residues. A vacuum vessel was used to safely transfer the sample from the glovebox to the XPS vacuum chamber, which ensured the minimal level of oxygen exposure and surface contamination.

## Conflict of interest

The authors declare no conflict of interest.

1

## Supporting information

As a service to our authors and readers, this journal provides supporting information supplied by the authors. Such materials are peer reviewed and may be re‐organized for online delivery, but are not copy‐edited or typeset. Technical support issues arising from supporting information (other than missing files) should be addressed to the authors.

Supporting Information

## Data Availability

The data that support the findings of this study are available from the corresponding author upon reasonable request.
