# Supplementary material for: Sodium Dual‐Ion Batteries with Concentrated Electrolytes
Source: ChemSusChem. 2022 Sep 26;16(4):e202201583. doi: 10.1002/cssc.202201583 (PMC10947385; doi:10.1002/cssc.202201583)
Supplement: Supplementary file 1 — Supporting Information [file CSSC-16-0-s001.pdf]

ChemSusChem

Supporting Information

Sodium Dual-Ion Batteries with Concentrated Electrolytes

Zhenyu Guo, Gang Cheng, Zhen Xu, Fei Xie,* Yong-Sheng Hu, Cecilia Mattevi, Maria-Magdalena Titirici,* and Maria Crespo Ribadeneyra*This publication is part of a collection of invited contributions focusing on “Dual-Ion Batteries”. Please visit to view all contributions.© 2022 The Authors. ChemSusChem published by Wiley-VCH GmbH. This is an open access article under the terms of the Creative Commons Attribution License, which permits use, distribution and reproduction in any medium, provided the original work is properly cited.

Results and discussion

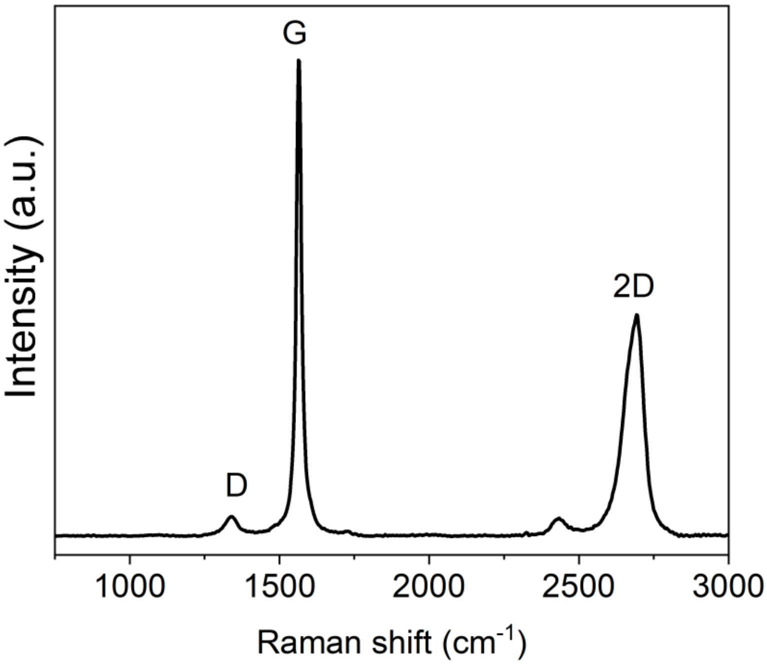

Figure S1 The Raman spectrum of the as-received pristine graphite powder, obtained via a 532nm laser.

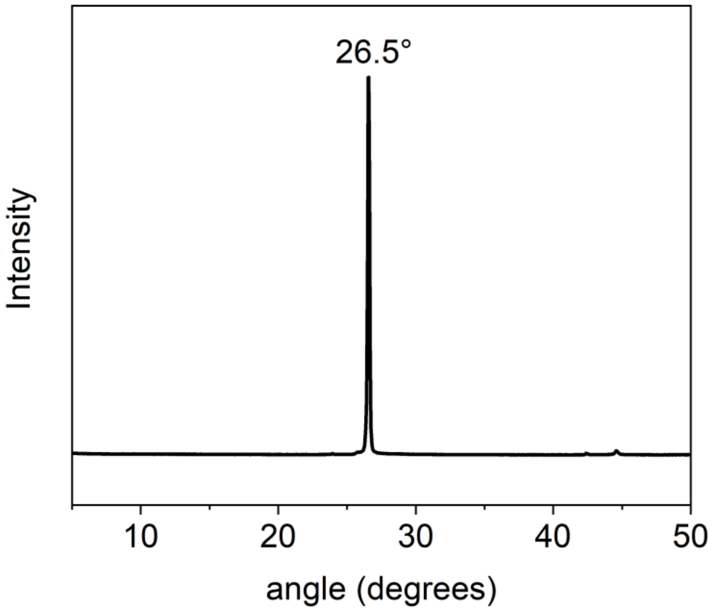

Figure S2 The XRD pattern of the as-received pristine graphite powder shows a high degree of graphitization and strong intensity of the (002) peak located at 26.5 degrees corresponding to the d-spacing of 3.35 Å.

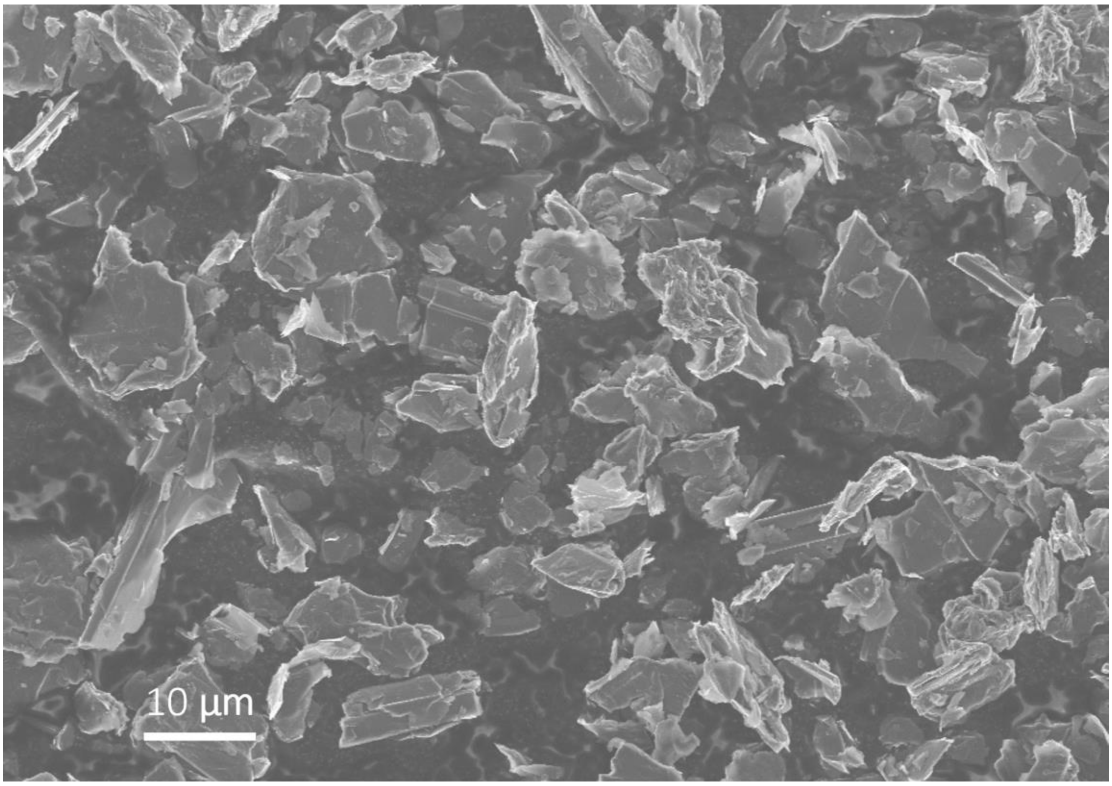

Figure S3 The SEM image of graphite powder.

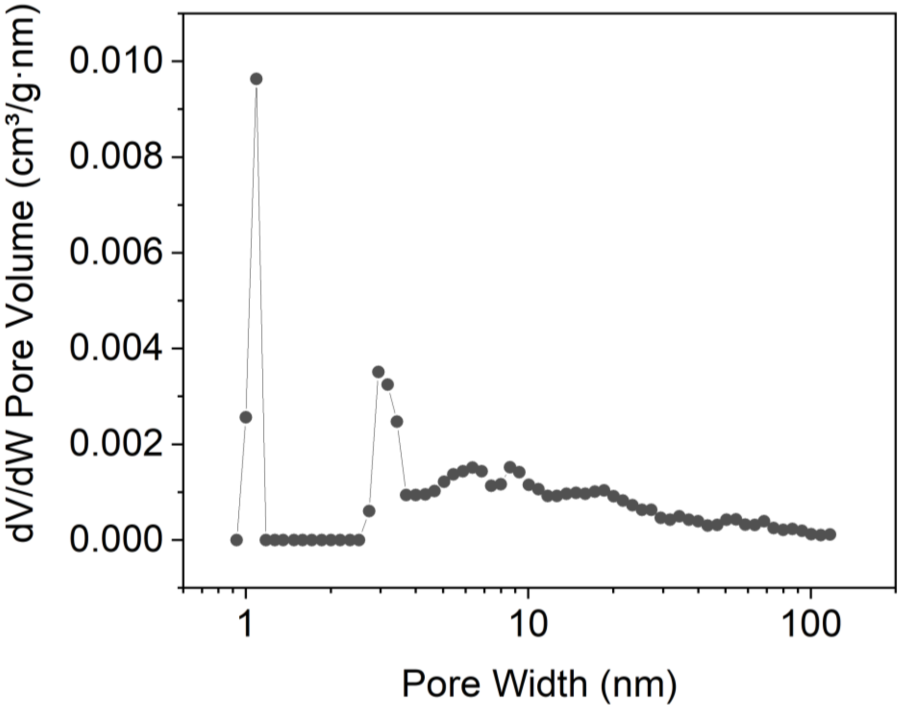

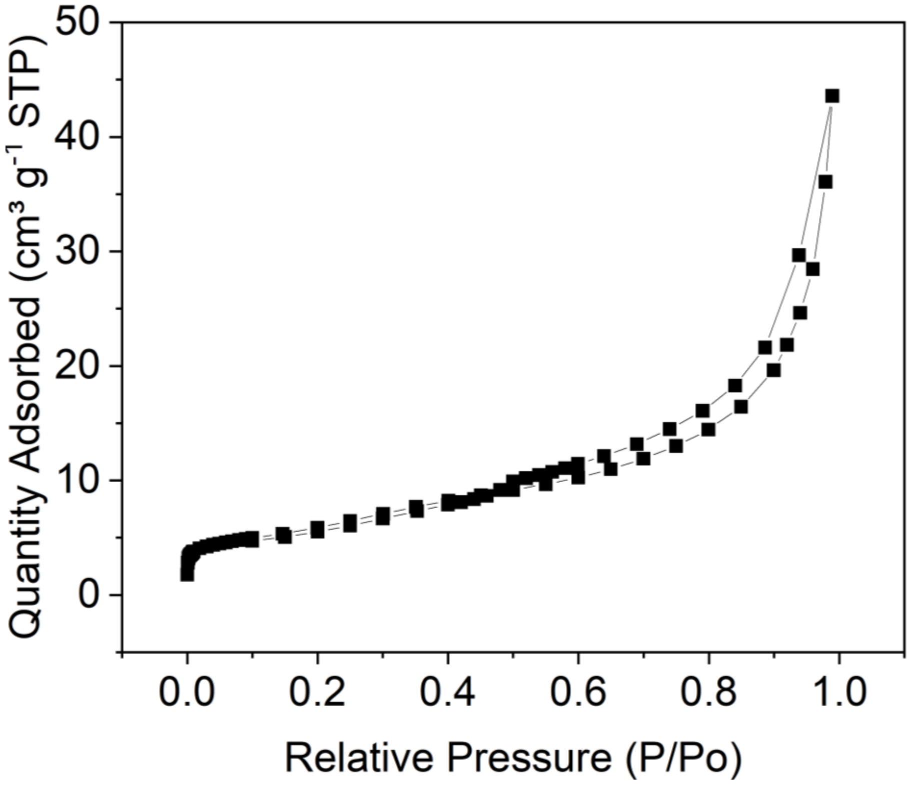

Figure S4 BET results of the graphite powder

Table 1 physical properties of the graphite powder

	I_D/I_G	002 peak from XRD	Interlayer Spacing (Å)	C at.%	O at.%	Surface area (m^2/g)	Size (μm)
Graphite	0.04	26.5°	3.33	96.15	3.85	21.8	9-10

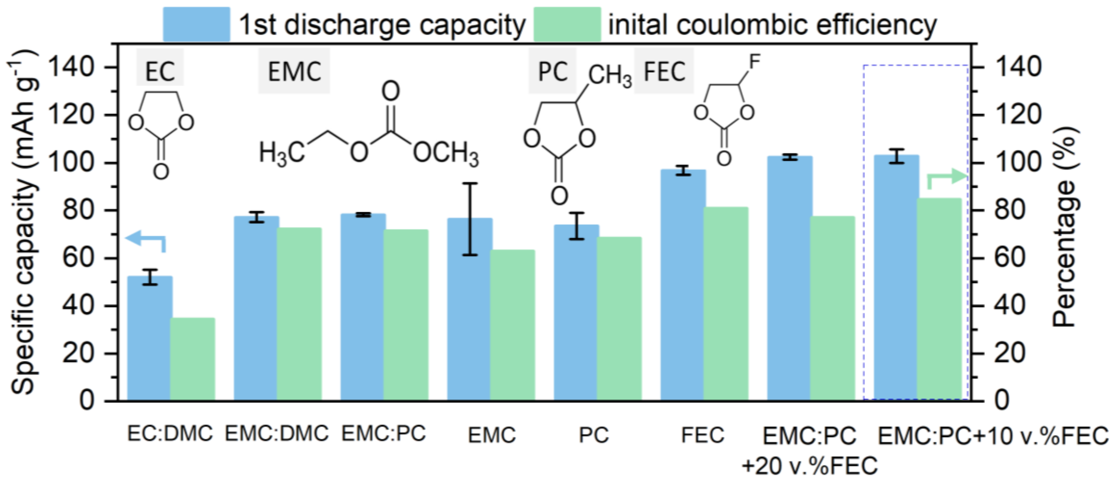

Figure S5 The averaged reversible capacity of the 1st cycle and the highest initial Coulombic efficiency of each solvent (three coin cells were assembled for each electrolyte).

Actually, we tested 5 coin cells using the electrolyte 1M $NaPF_6$ in EMC. The

electrochemical performance was very unstable, two of them failed before 5 V which also led to a huge error bar in the first discharge capacity.

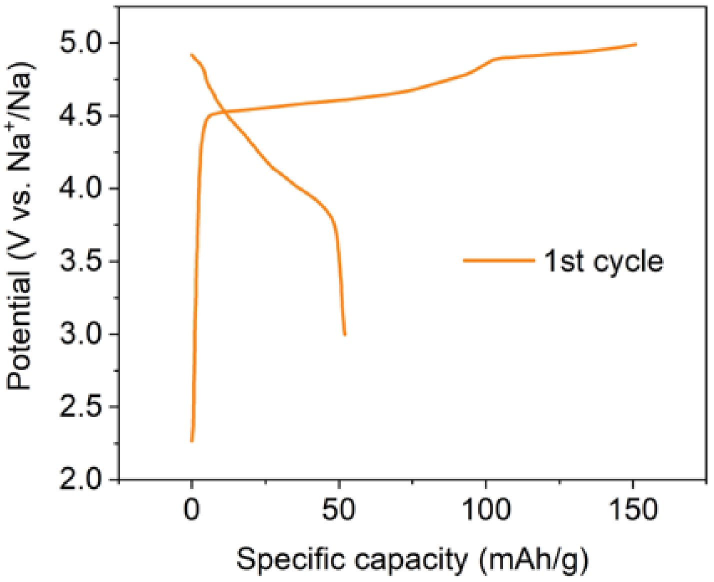

Figure S6 the first charge curve of Na |1M NaPF6 in EC: DMC|Graphite, showing no obvious charge plateau at around 4.8V.

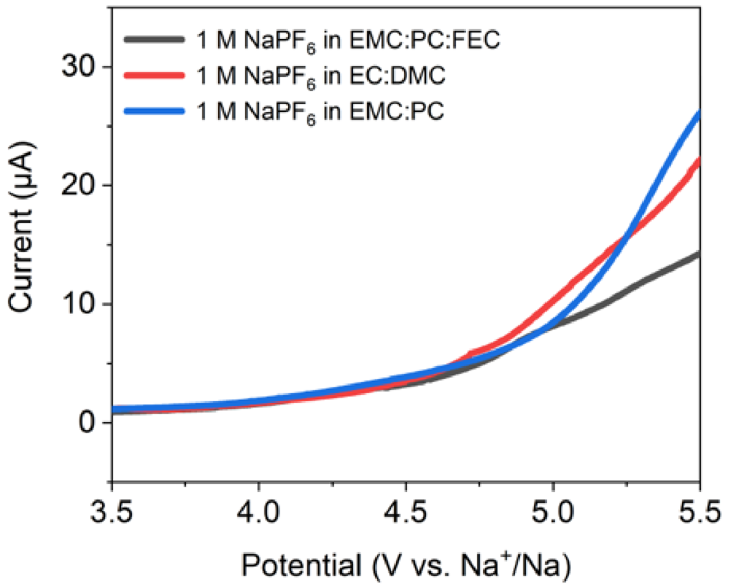

Figure S7 The stability test of different 1M $NaPF_6$ electrolytes.

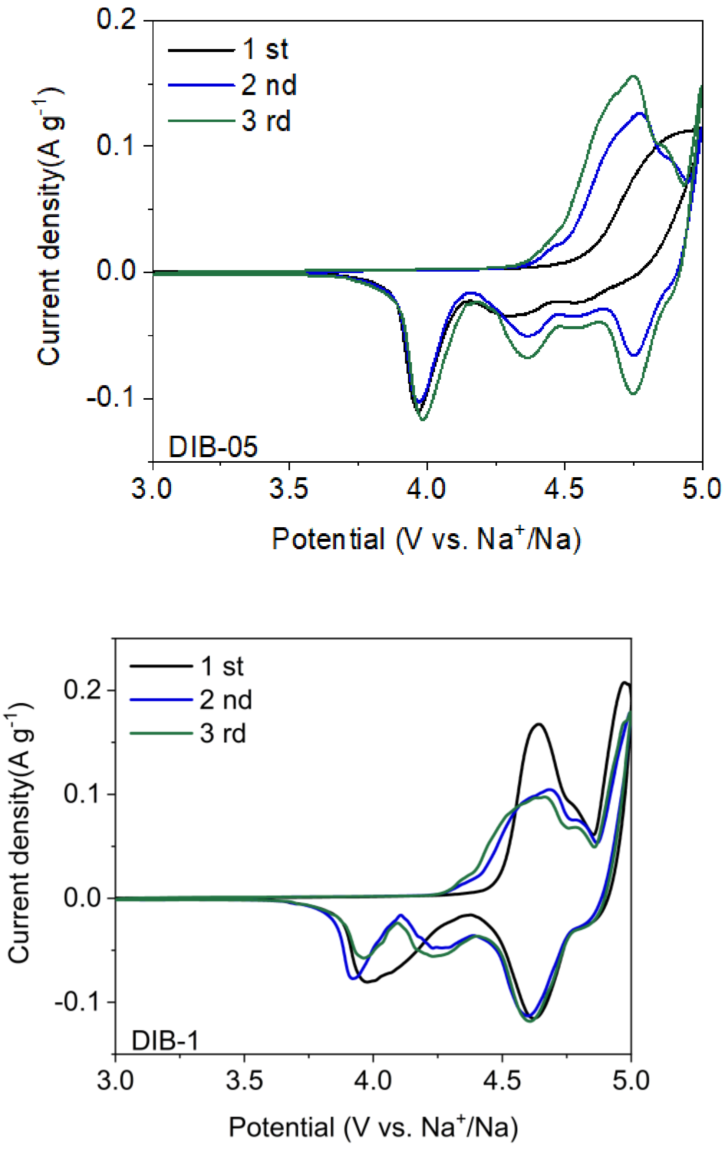

Figure S8 The first 3 cycles CV curves of DIB-1 and DIB-2 at 0.2 mV s^{-1}.

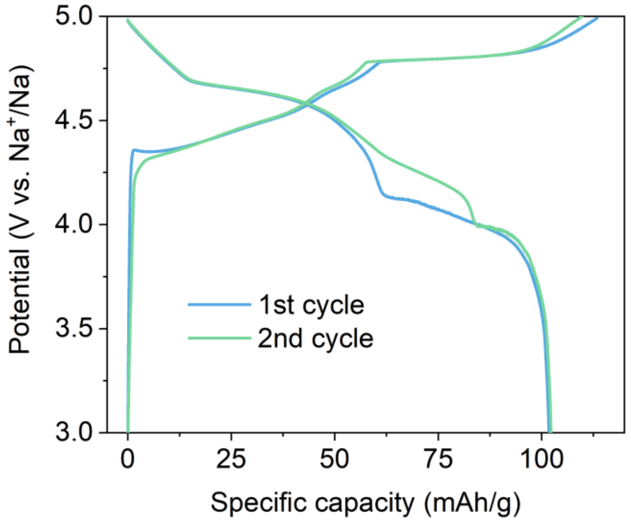

Figure S9 the 1st and 2nd cycles of DIBs in the 3M $NaPF_6$ in EMC:PC:FEC

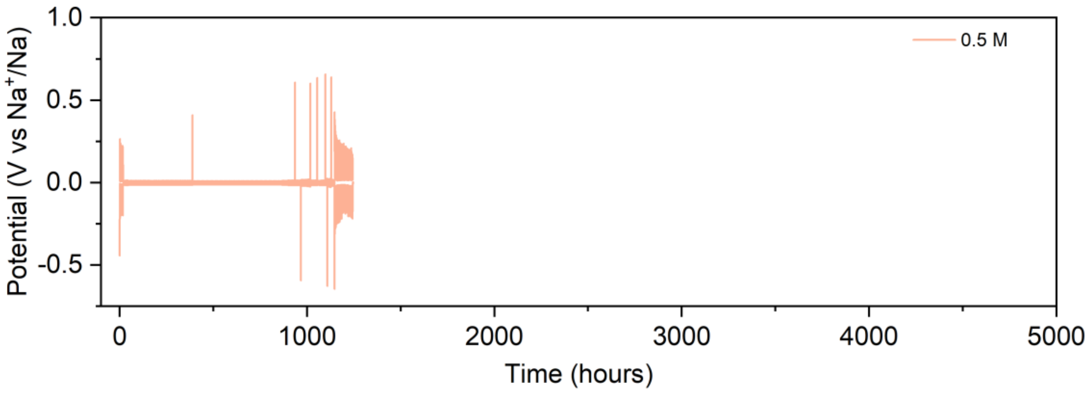

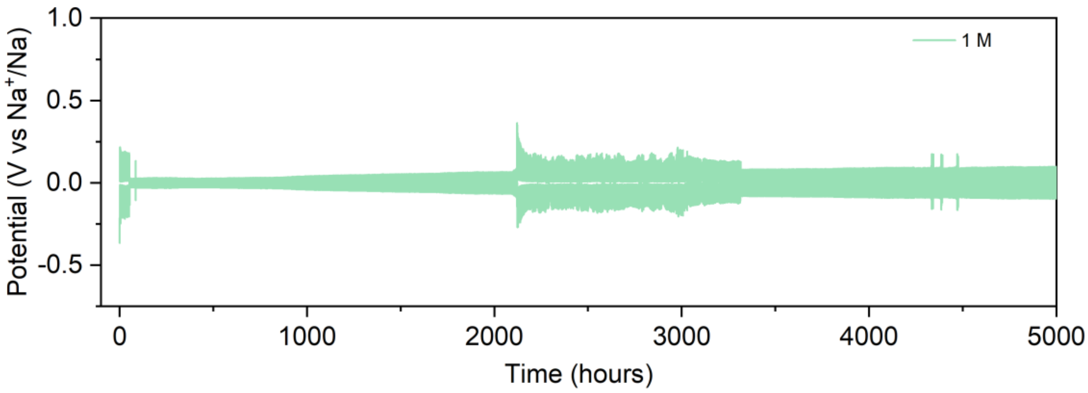

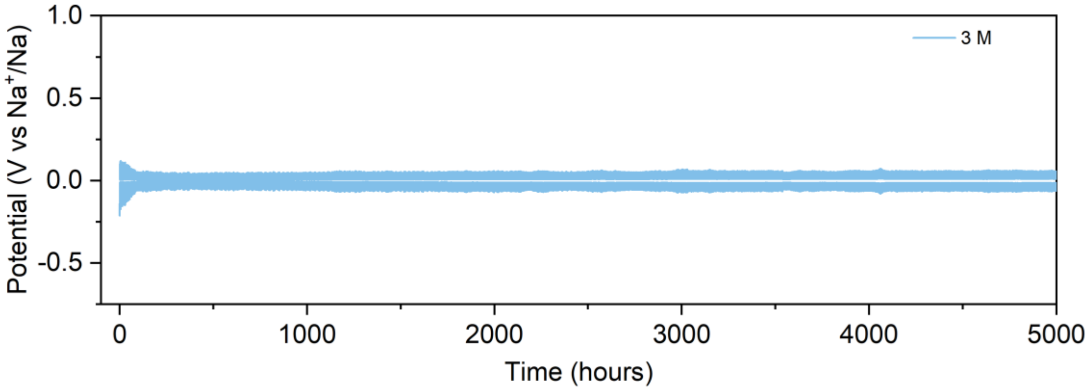

Figure S10 the Na||Na symmetric cells in 0.5 M, 1M and 3M $NaPF_6$ in EMC:PC:FEC

Table S2 the details percentage of each bond from XPS C1s

Bond	DIB-05(%)	DIB-1(%)	DIB-3(%)
C-C	35.48	28.65	37.98
C-O	33.67	34.34	31.71
C=O	12.31	17.92	13.95
C-F	12.4	10.83	9.89
$ROCO_2Na$	6.14	8.25	6.47

Table S3 the details percentage of each bond from XPS O1s

Name	DIB-05(%)	DIB-1(%)	DIB-3(%)
C-O	4.92	9.82	24.37
Na KLL	30.62	17.22	18.18
C=O	64.47	72.96	57.45

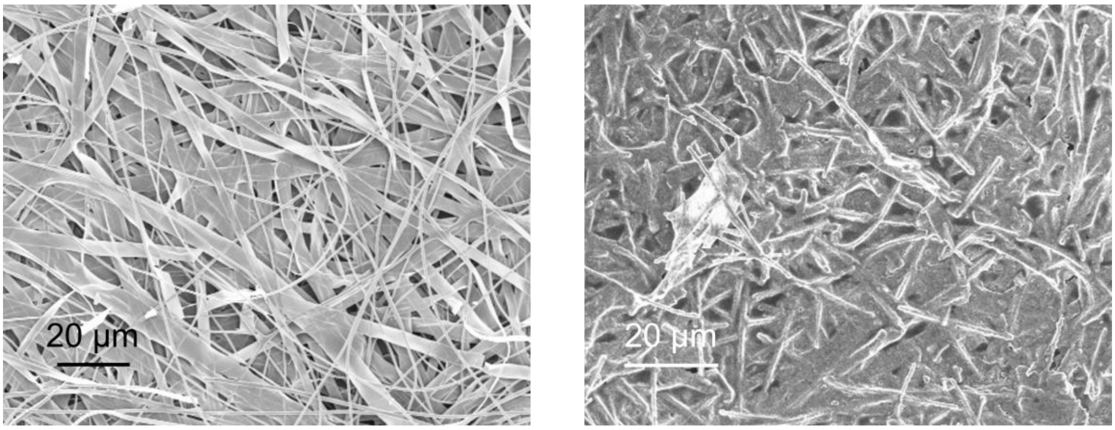

Figure S11 (left) the SEM image of the pristine carbon fiber mat; (right) the SEM image of the galvanostatically pre-plated carbon fiber mat morphology.
